# Alcohol industry involvement in science: A systematic review of the perspectives of the alcohol research community

**DOI:** 10.1111/dar.12826

**Published:** 2018-06-13

**Authors:** Jim McCambridge, Melissa Mialon

**Affiliations:** ^1^ Department of Health Sciences University of York York UK

**Keywords:** alcohol industry, bias, vested interests, science

## Abstract

**Issues:**

Alcohol companies have recently invested large sums of money in answering research questions to which they have clear vested interests in the outcomes. There have been extensive concerns about corporate influence on public health sciences, following the experience with the tobacco industry.

**Approach:**

This systematic review aims to investigate the perspectives of researchers on the activities of alcohol industry actors in relation to science, in order to guide future research. All data published in peer‐reviewed journals (including commentaries, opinion pieces, editorials and letters as well as research reports) were eligible for inclusion. This analysis focuses on the manifest rather than latent content of the articulated views, and accordingly adopts a thematic analysis using an inductive approach to the generation of themes.

**Key Findings:**

There are serious concerns identified in three main areas, principally defined by where the impacts of industry scientific activities occur; on evidence informed policy making (instrumental uses of research by industry actors), on the content of the scientific evidence base itself (industry funding as a source of bias); and on the processes of undertaking research (transgressions of basic scientific norms). There are also opposing views which provide a useful critique. The evidence‐base on the validity of all concerns has been slow to develop.

**Implications:**

The concerns are extensive, longstanding and unresolved and high quality investigations are needed.

**Conclusion:**

This study informs the detailed content of the research needed to address the concerns identified here.

## Introduction

Approximately two‐thirds of the funding for a recently commenced $100 million dollar clinical trial to investigate possible cardiovascular benefits of alcohol has been reported to have been provided by five global alcohol producers 1. The scale of this investment in answering research questions in which the donors have clear vested interests is striking, as there is no known precedent in alcohol industry funding of research. This development arises against the backdrop of longstanding discussions within the research community about alcohol industry involvement in science 2, 3. There are also wider concerns about corporate influence on public health sciences and policy 4, 5 and funding effects on research findings 6, 7.

The significance of the tobacco industry's attempts to shape science as a means of influencing policy became apparent after the mandated release of internal company documents 8. Bero 9 identified six strategies used by the tobacco industry to shape the evidence on risks associated with tobacco smoking: (i) Fund research that supports the interest group position; (ii) Publish research that supports the interest group position; (iii) Suppress research that does not support the interest group position; (iv) Criticise research that does not support the interest group position; (v) Disseminate interest group data or interpretation of risk in the lay press; and (vi) Disseminate interest group data or interpretation of risk directly to policy makers 9. Later comparative study suggested similar strategies have also been used in other industries where internal documents are also available 10. There have also been historical investigations spanning decades which show environmental sciences being undermined by corporate vested interests using similar methods 11.

In this context, this systematic review aims to investigate the nature of the attention given by alcohol researchers to the activities of alcohol industry actors in relation to science. No previous systematic review in this area has been conducted. This study is undertaken to develop hypotheses to guide future research on the alcohol industry and science. Specifically, this study seeks to: (i) identify issues raised in peer‐reviewed journals on alcohol industry involvement in science; and (ii) analyse these issues with a view to ascertaining what empirical research may contribute.

## Methods

This study examines how the research community has considered alcohol industry involvement in science. It was anticipated at the outset that there would be few dedicated studies, so this review is designed to synthesise existing perspectives as a preliminary investigation. The study focuses on the manifest rather than latent content of the articulated views, and accordingly adopts a thematic analysis using an inductive approach to the generation of themes 12. This approach has been described as being particularly well suited to appreciation of a given group's conceptualisation of a phenomenon being studied 12.

### *Data collection*

To be included in this review, information contained in peer‐reviewed journals (including in commentaries, opinion pieces, editorials and letters as well as research reports) must:

- Focus on the activities of alcohol industry actors in relation to science;
- Be published during the period 1980–2016 inclusive;
- Be published in English.

The earliest date is chosen to include data prior to the global concentration of alcohol producers since the 1990s 13. No grey literature is included in this systematic review. Literature search strategies were developed using both MeSH terms and key words. Eight health, social science and business databases were searched:

- Web of Science Core Collection;
- BIOSIS Citation Index (Web of Science interface);
- CINAHL Plus (EBSCOhost interface);
- Business Source Premier (EBSCOhost interface);
- Embase (Ovid interface);
- MEDLINE (Ovid interface);
- PsycINFO (Ovid interface);
- Scopus (Scopus interface).

The basic search strategy was organised around the three constructs of ‘alcohol’, ‘industry’ and ‘science’ and developed with the support of a specialist librarian. The search strategy for MEDLINE is presented in Appendix S1 (Supporting information). Searches were conducted on the 27 February 2017. The material retrieved was downloaded and imported to EndNote. Duplicates were removed using this software. Titles (and abstracts where available) were screened by MM, with a random sample checked by JM (10% of all data). Potentially eligible full texts were obtained, usually in PDF format. Eligibility was determined by both authors separately, with any disagreements resolved through discussion. Hand searching of the journals *Addiction* and *Alcohol and Alcoholism* was undertaken. We also contacted topic experts (identified from those who have published included material) with a view to including additional data sources that may have been missed. We did not publish a protocol for this review.

### *Data analysis*

We began by reading all included material. Data were extracted from all included records by both authors using direct capture of relevant text from materials via cut and paste with Microsoft Word and NVivo. Both authors coded all data separately at first, then met to discuss preliminary themes and their labelling, and subsequent rounds of coding. The intention was to make this process as inductive as possible, eschewing the use of any pre‐existing analytic categories.

The analyses explored thematically the research community's views on alcohol industry involvement in science, summarising and interrogating identified issues with a view to providing an interpretive synthesis. This involved consideration of how far the issues raised have been studied, and what any such studies have found, without making any assumptions about the validity of the issues raised. Themes were iteratively refined, with revisions of labels and content as the analysis progressed, staying close to the data 12.

The nature of this study precluded formal assessment of risk of bias as the information sought is intrinsically biased in the sense that it does not necessarily result from any scientific process. Both authors have studied corporate actors and public health, and the first author has specifically investigated the alcohol industry, thus having publications included within this review.

We offer a necessarily parsimonious account of a large dataset, reporting only on issues that have been prominently discussed. We cite included material to permit appreciation of how widespread particular views are. Quotations are used to provide direct access to the most informative data. The analyses are thus designed to present the most salient data, which should be straightforwardly recognisable and credible to the participants in these debates.

As a member validation exercise, we sent a draft copy of the results to eight researchers, four each to those with concerns and those who were critical of these concerns or their handling (see Results), and asked three questions about fairness, missing data and concerns about data handling. We received comments from seven, with comments from six endorsing the fairness of our approach and data handling, containing respondent views on the issues studied, and/or making helpful suggestions, including two who were critical of the concerns. One respondent, however, found the tone ‘adversarial and accusatory’ and viewed the unequal number of pages devoted to both sides of the debate as a ‘possible indicator of the article's bias’. Interestingly, the respondent suggested that the concerns ‘perspective may be oversimplified’ rather than seeing the debate as oppositional in character (see ‘Opposing views to articulated concerns’ section in Results). We reviewed the data and our reporting and decided not to alter the Results section on the basis of comments received from all 7 respondents. We also judged that it was appropriate to report the view expressed here that was different.

## Results

**Figure 1:**
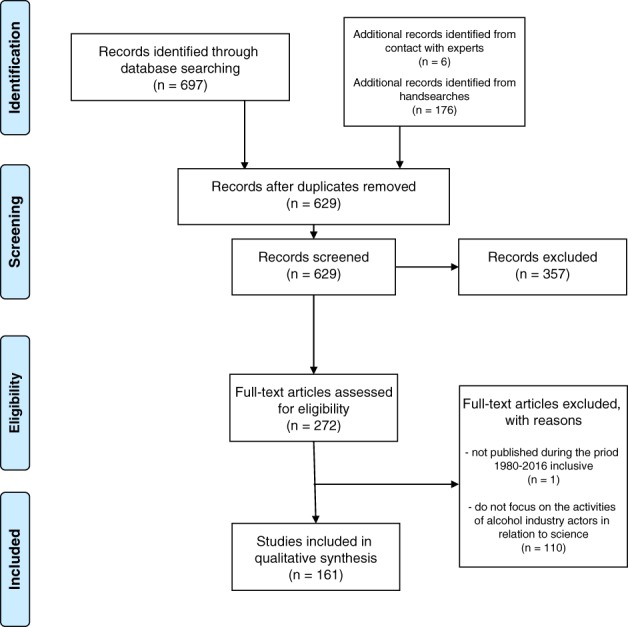
PRISMA flowchart.

The PRISMA flowchart is presented in Figure 1, with 161 records included. There are serious concerns articulated in the bulk of the attention given by the research community to alcohol industry activities in science, with some scientists expressing contrary views. There are three main thematic areas of concern, principally defined by where the impacts of industry scientific activities occur; on evidence informed policy making, on the content of the scientific evidence base itself and on the processes of undertaking research.

### *Instrumental uses of research by alcohol industry actors*

#### *Strategic motivations*

A core concern of the research community relates to the reasons why alcohol industry actors are involved in science 14, 15, 16, 17, 18, 19, 20, 21, 22, 23, 24, 25, 26, 27, 28, 29. Attention has been consistently drawn to the legitimation and public relations benefits of industry involvements in science. Babor 20 provided an example of the chief executive of a global alcohol producer articulating a strategic motivation for providing research funding to a university (in a speech to an industry group reported in a national newspaper):

‘*He* [the chief executive] *said the company did not want problems with binge drinking to lead governments to place higher taxes on its products and thus eat into revenues. The UCD* [an Irish university] *research funding is thus the perfect example of “enlightened self‐interest*”’ 20.

It has been widely suggested that there is an inherent conflict of interest between the commercial goals of industry actors and the production and dissemination of public health research 2, 3, 16, 21, 26, 29, 30, 31, 32, 33, 34, 35, 36, 37, 38, 39, 40, 41. This arises because such research may provide evidence of the harms caused by the industry's products and practices 17, 26. Cook 22 proposed that evidence demonstrating conflict of interest arises from how existing evidence is interpreted by industry actors:

‘*That they* [the alcohol industry] *evaluate the evidence in the way that they do therefore appears to many impartial observers to be a manifestation of, or evidence for, the existence of the very conflict of interests that they deny*’ 22.

#### *Policy influence*

The possible value of relationships with the international research community for influencing policy makers has been of particular concern 2, 3, 20, 29, 30, 31, 42, 43, 44, 45, 46, 47, 48, 49, 50, 51, 52, 53, 54, with the International Center for Alcohol Policies (ICAP), an alcohol industry organisation created by major global producers being prominent 18, 46, 55, 56, 57, 58, 59, 60, 61, 62, 63, 64, 65, 66, 67, 68, 69, 70. ICAP activities in low and middle income countries have received widespread attention 18, 57, 58, 62, 64, 69, 71. As Jernigan and Mosher elaborate; ‘collaboration of scientists with ICAP provides the credentials the industry needs to convince governments of developing countries to neglect or weaken environmental safeguards regarding alcohol use’ 57.

Industry actors collectively fund such organisations which claim to be concerned with the social aspects of the harms done by alcohol that are; ‘distanced from obvious corporate interests by funding “not‐for‐profit” organisations with innocuous names’ 46 like ICAP. Tobacco control researchers refer to such organisations as ‘front groups’ 72. Anderson suggested that ‘a key work of social aspects organizations is to gain credibility and respectability through recruiting scientists’ 15.

Scientific meetings, conferences and publications are prominent among concerns 46. According to Babor and Robaina 3;

‘*ICAP has sponsored 12 other conferences, involving a mixture of industry representatives, academics, and government officials, in Europe, Africa, and Asia between 2003 and 2006… ICAP has published 10 books in its Alcohol and Society series, most dealing with scientific and public policy issues. The books tend to be co‐authored or coedited by a combination of ICAP staff, academic researchers, and industry representatives*’ 3.

Babor and Xuan 73 described shortcomings of an ICAP report which; ‘seems to present conclusions that are inconsistent with its own data or unwarranted because of faulty survey methodology. The conclusions are also inconsistent with the considerable body of policy research that has been published in recent years’ 73. Room 74 identified dissemination of research findings in line with vested interests more generally to be the case for social aspects organisations; ‘measures of undoubted effectiveness which would impair the industry's collective interest are simply left off the table’ 74.

In a dedicated study by Jernigan 58, ICAP appears to function specifically to counter the work of the World Health Organization, with literature reviews and other publications including model alcohol policies congenial to industry interests, and whose contents; ‘excluded or attempted to refute evidence regarding the most effective strategies to reduce and prevent alcohol‐related harm’ 58. Alongside lack of information on methods, the extremely biased content of literature reviews is emphasised 58.

These findings are similar to another study which identifies serious misrepresentations of evidence in submissions made to influence policy in Scotland 75. According to Hawkins and McCambridge 76, the cumulative impact of such literature reviews and other reports are to yield; ‘a methodologically flawed and highly biased but internally consistent parallel literature to the international peer‐reviewed scientific literature’ 76.

Critical attention has been directed at other industry‐funded books and reports which have apparently been commissioned for the purposes of policy influence. For example, Edwards and Savva 77 drew attention to the lack of any declaration of industry sponsorship in one book. Alcohol industry‐funded think tanks have collaborated with, or commissioned reports from, academic sources which appear designed to influence policy debates 76, 78. For example, Jackson and Kypri 79 critiqued a report by an anthropologist funded by a New Zealand alcohol producer which reached the unlikely conclusion that alcohol consumption is not responsible for night‐time violence.

This review has identified, however, only a small number of research studies of instrumental uses of research, including those pertaining to social aspects organisations and activities at the science/policy interface (see Table 1).

**Table 1:** Research studies† included within this systematic review

Study	Stated aims	Study design	Key author findings	Notes on risk of bias
Babor and Xuan 73	‘Compare two (…) examples of survey research published in the grey literature’, one published by the World Health Organization and the other by the International Center for Alcohol Policies, a social aspect organisation funded by the alcohol industry.	Documentary analysis.	‘Although the two studies share a similar survey methodology and common policy aims, the findings and conclusions are very different’. (…) ‘In the case of the ICAP survey, we have identified significant methodological and inferential flaws in the current report that would preclude meaningful comparisons with data collected in the future’.	The designs of both surveys mean that study findings are susceptible to multiple forms of bias. The ICAP survey reaches conclusions which the authors of this study note are inconsistent with its own data. The documentary analysis identifies key biases, though the nature of this study means that an assessment of risk of bias would require examination of the survey reports, for example, to address comprehensiveness.
Babor 2	‘Evaluate the ethical, professional and scientific challenges that have emerged from industry involvement in alcohol science’.	Narrative review.	‘Industry involvement in alcohol science was identified in seven areas: (i) sponsorship of research funding organisations; (ii) direct financing of university‐based scientists and centres; (iii) studies conducted through contract research organisations; (iv) research conducted by trade organisations and social aspects/public relations organisations; (v) efforts to influence public perceptions of research, research findings and alcohol policies; (vi) publication of scientific documents and support of scientific journals; and (vii) sponsorship of scientific conferences and presentations at conferences’.	This study gathers and evaluates data from a wide range of different primary and secondary sources. As such it is not designed in ways that permit direct assessment of risk of bias. This study offers a key statement of possible targets for future research. These comments apply also to the later update of this study by Babor & Robaina (2013).
Jernigan 58	‘Document strategies used by alcohol producers to influence national and global science and policy’.	Case study.	‘Their strategies include producing scholarly publications with incomplete, distorted views of the science underlying alcohol policies; pressuring national and international governmental institutions; and encouraging collaboration of public health researchers with alcohol industry‐funded organisations and researchers’.	This study gathers and evaluates data from a wide range of different primary and secondary sources. As such it does not lend itself to direct assessment of risk of bias. This study offers a key statement of possible targets for future research and monitoring.
Babor and Robaina 3	‘[Explore] the emerging relationships among the alcohol industry, academic medicine, and the public health community in the context of public health theory dealing with corporate social responsibility’.	Narrative review.	‘The alcohol industry has intensified its scientific and policy‐related activities under the general framework of corporate social responsibility initiatives, most of which can be described as instrumental to the industry's economic interests’.	This study gathers and evaluates data from a wide range of different primary and secondary sources. As such it is not designed in ways that permit direct assessment of risk of bias. This study offers a key statement of possible targets for future research. These comments apply also to the earlier version of this review by Babor (2009).
McCambridge *et al*. 75	‘Examine how research evidence is used in alcohol industry submissions made to a Scottish Government consultation in 2008 to advocate policies in line with their commercial interests’.	Documentary analysis/case study.	‘Industry actors consistently oppose the approaches found in research to be most likely to be effective at a population level without actually engaging with the research literature in any depth. Strong evidence is misrepresented and weak evidence is promoted. Unsubstantiated claims are made about the adverse effects of unfavoured policy proposals and advocacy of policies favoured by industry is not supported by the presentation of evidence’.	This study analyses data from a clearly identifiable set of documents pertaining to a globally important juncture in national alcohol policy development. There are few details provided of the analytic methods used, or of any conceptual framework used to interpret the results. Assessment of risk of bias draws attention to these reporting limitations.
Hawkins and McCambridge 76	‘[Examine] how SABMiller engaged the think tank Demos to produce reports on binge drinking, which were heavily promoted among policymakers at crucial stages in the development of the UK government's 2012 alcohol strategy’.	Case study.	‘The perceived independence of an influential think tank was used to promote industry interests in tactics similar to those of transnational tobacco corporations’.	This case study examines particular reports produced by a think tank funded by an alcohol company, and focuses on the events surrounding their publication, within the context of a controversial period in alcohol policy. The study relies on multiple data sources that are triangulated, though there is little theoretical data provided. The study design does not lend itself to direct assessment of risk of bias.
McCambridge 85	‘[Examine] whether findings of alcohol's protective effects on cardiovascular disease may be biased by industry funding’.	Secondary meta‐analysis of systematic review data.	‘This study gives no specific grounds for concern that alcohol industry funding has biased what is known about the protective effects of alcohol on cardiovascular disease, apart from with regard to stroke. Our investigation provides evidence that findings from studies evaluating associations between alcohol consumption and incident stroke vary considerably according to whether or not there is concern about industry funding’.	The systematic review context of this study provides various key assurances about risk of bias. There are specific concerns, however, declared by the authors about the adequacy of the correspondence and online data collection methods used for identification of industry funding. This is quite likely to be biased in ways which are complex, through incomplete identification of funding.
Avery *et al*. 119	‘Examine all alcohol industry submissions …to assist in understanding how those with vested interests contribute to policy development’.	Documentary analysis.	‘Alcohol industry submissions sought to undermine community concern, debate the evidence, promote ineffective measures which are no threat to the profit margins and attack independent health professionals and researchers’.	This study analyses data from a clearly identifiable set of documents pertaining to an important alcohol policy issue. Appropriate analytic methods are used, and find little depth to the data presented. There are data adequacy and generalisability concerns.
Jackson 79	‘A critique [of a report commissioned by the alcohol producer Lion Pty Limited, which consisted of an anthropological investigation into aggression and violence], addressing key claims with reference to the scientific evidence’.	Documentary analysis.	‘In our view, the report lacks credibility as a piece of independent academic research, failing to present a balanced appraisal of the relevant literature. Dr. Fox overstates the effectiveness of liquor accords, social marketing and alcohol education and underplays the causal role of alcohol in violence’.	This study analyses a specific industry‐funded report designed to influence policy in the context of the scientific literature. There is not a statement of the analytic methods used. This study is not designed in ways that lends itself to straightforward assessment of risk of bias.

† Studies are defined as reports of data collection or analysis designed to address specific research questions or objectives.

### *Alcohol industry funding as a source of bias*

In the first narrative literature review on the subject of alcohol industry activity in science, Babor 2 identified the following as concerns, almost all of which relate to use of funding: (i) Sponsorship of research funding organisations; (ii) direct financing of university‐based scientists and centres; (iii) studies conducted through contract research organisations; (iv) research conducted by trade organisations and social aspects/public relations organisations; (v) efforts to influence public perceptions of research, research findings and alcohol policies; (vi) publication of scientific documents and support of scientific journals; and (vii) sponsorship of scientific conferences and presentations at conferences 2.

The nature of bias induced by research funding is considered here to have deleterious impacts at the levels of the individual research study 2, 3, 16, 17, 21, 26, 32, 36, 59, 66, 67, 73, 74, 80, 81, 82, 83, 84, 85, 86, 87, 88, 89, 90, 91, 92. Bias could also have broader adverse impacts on research topic areas, research agendas, individual researchers, as well as institutions such as universities, academic conferences and societies and journals 2, 3, 15, 16, 18 25, 26, 29, 37, 38, 41, 42, 46, 51, 52, 56, 59, 61, 64, 65, 66, 78 80, 81, 87, 89, 93, 94, 95, 96, 97, 98, 99, 100, 101, 102, 103, 104, 105.

#### *Funding effects on research studies, topic areas and research agenda setting*

Smith suggested that; “bias is subtle and pervasive” 91 in funding effects, and by extension that the mechanisms are complex and may be difficult to detect. McCambridge and Hartwell 85 noted; ‘the lack of prior quantitative study of the alcohol industry's possible involvement in subversion of science is somewhat surprising in view of the evidence available on the effects of funding by other industries’ 85. Andreasson and McCambridge 16 suggested that; ‘in addition to the potential for bias in the design, conduct, and reporting of research, industry funding for a particular topic may or may not be selected for scientific reasons. To the extent that funding for a particular research question is dictated by commercial reasons, it is likely to alter the research agenda’ 16. Other scientists also address cumulative bias on topic areas and resulting agenda setting 25, 46, 56, 80, 86, 99. According to Stein 37, ‘industry only provides funding for certain kinds of issue, and fails to address many key research and policy questions’ 37. It should be remembered that these data are expressed views, rather than the findings of empirical research.

#### *Biasing the work of individual researchers*

A 1985 *BMJ* editorial noted the potential of research funding to*; ‘*buy off an influential and articulate opponent’ 21 attesting to blunt impacts on individual researchers. Babor and Miller 32 described that; ‘conflicts of interest can further influence behaviour by imposing a “sense of indebtedness”, and thereby the obligation to reciprocate’ 32. Babor and Robaina 3 placed this concern within career contexts; ‘investigators who receive funds are typically at an early stage of their medical or scientific careers’ 3.

Catford 81 noted the capacity of funding to; ‘create a dependence on this form of research funding which may then inhibit other independent research and inquiry’ 81, which together with the psychological effects suggests the possibility of cumulative impacts on individual researchers over time, presumably which only become serious for a small minority of all recipients of funding. Such effects on individuals may be considered to reinforce, and be reinforced by, cumulative bias impacts on topic areas and research agendas.

#### *Funding effects on institutions: universities, journals and academic societies*

Attention has been drawn to the possible effects of funding on a range of institutions, including the blurring of institutional boundaries. For example, Pinsky and Laranjeira 47 have described how in Brazil; ‘the industry has also begun recruiting alcohol researchers into its ranks by donating funds to an apparently independent university‐based non‐governmental organization (NGO)’ 47. Similarly, Babor and Robaina 3 identified a close relationship between; ‘the International Scientific Forum on Alcohol Research…an undertaking of Boston University's Institute of Lifestyle and Health jointly with Alcohol in Moderation, a UK [social aspects] organization’ 3. They suggest that; ‘industry funding has become a contentious issue at some universities…because of the potential for conflict of interest’ 3. Others have suggested possible funding effects on prestigious universities 16, 58, 76.

Babor *et al*. 17 reported on the resignation of the editor of the journal *Alcohol and Alcoholism*, citing interference by industry funders in the editorial work of the journal, which at the time was supported by industry funding. Babor and Robaina 3 identified how industry payments to journals to produce special issues can become vehicles of dissemination.

There are a range of concerns articulated about industry funding of academic conferences, in addition to those events organised by industry actors themselves. For example, Saitz 101 pointed out that the Research Society on Alcoholism meetings, the world's largest scientific conferences on alcohol, are supported by industry funding. Conference organisers and academic societies in receipt of industry funding such as the International Council on Alcohol and Addictions 106 and the International Society of Addiction Medicine 107 have defended their procedures against suggestions of bias.

This review identifies only three studies of bias in academic research comprising two narrative literature reviews of the various issues covered in this section 2, 3, and one meta analytic study 85. See Table 1 for details of main findings.

### *Other transgressions of basic scientific norms*

One alcohol policy scientist summarised the criticism received from various industry actors as follows: ‘they [the alcohol industry] are engaging with science but not with the scientific process ‐ peer review, constructive discussion, balanced arguments, debate, critical appraisal of the totality of the evidence ‐ so we're just responding to the same points over and over again’ 78. There are more specific expressions of concerns about impacts on the processes of science 2, 3, 17, 20, 32, 44, 73, 74, 76, 78, 84, 98, 107, 108, 109.

#### *Deficits in peer review*

Babor 20 described the award of substantial research funding to a research group by one alcohol company apparently without any peer review process. Foxcroft 84 agreed to peer review an ICAP report and found the process inadequate. Babor 2 noted that an application to the alcohol industry's dedicated research funding organisations; ‘requires only a few pages of detail, in contrast to the extensive application process demanded by governmental funding agencies that support alcohol research in Europe and America’ 2, calling into question the rigour of the decision‐making process. Various authors 2, 76, 79 identify the conduct of studies without ethical approval.

#### *Lack of transparency*

There is a long history of lack of disclosure of industry funding by researchers. Jones 98 reminds us that the inventor of the Student's *t*‐test; ‘the statistician and chemist W. S. Gosset published important papers under the pseudonym “Student” because of various links he had with an industrial company, namely the Guinness brewery’ 98. This issue has given cause for concern for more than 20 years 17, 110. More recent concerns about lack of disclosure by researchers continue to be articulated 2. This is despite the introduction of gradually more stringent standards for disclosure of conflicts of interest, both in addiction journals 111 and in the wider journal publishing sphere 112, 113. Babor and Miller 32 described various difficulties in enforcing such policies among those who are reluctant to adhere to normative standards; ‘The authors argued that the ICAP connection was not relevant to the topic of their research…A check of the organizations with which the authors were involved revealed a convoluted set of financial connections’ 32. They went on to suggest that disclosure procedures may not be achieving the intended purpose of promoting transparency 32. Here again, social aspects organisations seem to play an important role, as demonstrated by the numerous references to the reports produced by ICAP 2, 3, 20, 73, 74, 84, 108, 114.

#### *Lack of trust*

Room drew attention to the ways in which industry actors presented themselves; ‘not as a representative of private corporate interests, but rather in two other guises. One is as a non‐profit scientific entity acting on behalf of the common public good… The other guise is as a go‐between or broker between public and private interests…as the “third party” bringing together private and public interests in a “global partnership”’ 115.

According to Raw 65, for researchers inclined to work with alcohol industry actors, the risks involved centred around a lack of trust;

‘The obvious danger of working in partnership with them is not being quite sure whose agenda you are working to, or whether the industry agenda is quite what they would like you to believe it is…the alcohol industry appears to have decided (like the tobacco industry) not to work with the independent public health science field, and instead develop their own disinformation research agenda. I think this makes it difficult to trust them’ 65.

#### *Uncollegiate attacks on researchers and research*

Research that conflicts with vested interests is also attacked 2, 3, 17, 20, 24, 30, 44, 96, 116, 117. A repeatedly cited example 2, 3, 17, 31, 43, 70, 91, 92, 96, 99, 118, is a case revealed by a whistle‐blower, involving an alcohol industry organisation, the Portman Group, in the year prior to the formation of ICAP. It sought to pay five academics large fees to produce anonymous critiques of a major World Health Organization summary of the alcohol policy evidence‐base 91. When writing to these academics, the director of the Portman Group suggested; ‘I do not think that this book can be allowed to go unchallenged… it is in my opinion, unsatisfactory in the way it selects the evidence and draws conclusions from it. Results are reported and research cited selectively. Evidence which does not support the views expressed is frequently ignored. But I am far from an expert’ 91. This request was designed to undermine the consensus position statement of the world's leading alcohol policy scientists. Gmel *et al*. 44 describe a commissioned attack on their work by an academic without subject expertise presented at a press conference. There was also evidence of attacks on researchers in a study of submissions made to a public consultation 119.

#### *Similarities in science involvements across industries*

Parallels are drawn between apparent similarities in the tactics of the alcohol industry and of other industries, most notably the tobacco industry 18, 24, 34, 54, 58, 85, 96, 120, 121, 122. To explain this, Jernigan identified the influence of tobacco company ownership of a major beer producer in the establishment of ICAP 58.

### *Opposing views to articulated concerns*

As might be expected, there are a range of views within the research community about the issues analysed here. In contrast to the various concerns discussed previously, there are also opposing views which are much less frequently articulated but may provide material with which to question the validity of those concerns 27, 55, 106, 107, 123, 124, 125, 126, 127, 128, 129, 130, 131, 132, 133, 134, 135, 136, 137, 138, 139, 140, 141, 142, 143, 144, 145, 146, 147, 148, 149, 150, 151.

#### *A simplistic and unhelpful dualism*

The most commonly expressed views are that the concerns are over simplistic and create a moralistic dichotomy, containing value judgements that are not made explicit. This is succinctly articulated by Chafetz 124 as a ‘good guys—bad guys’ narrative. Davies and Rotgers identify an implication; ‘that some research (“virtuous” research) is value‐free, uncompetitive and not conflicted, whereas “commercial” research is uniquely plagued by those things’ 127. Davies and Rotgers go on to articulate that such an approach is inherently divisive; ‘we fear an increasing number of witch‐hunts involving people whose research we do not like for ideological reasons that have nothing to do with their science’ 127. Fillmore and Roizen 55 regard the concerns as a ‘campaign to marginalize beverage industry involvement in alcohol science’ 55.

#### *Controversies and doubts*

Concerns about industry funding or other behaviour often centre on episodes of controversy (see Table 2). Some researchers regard the risks or problems as being over‐stated, and cite their own experience of unproblematic alcohol industry funding as evidence 133, 135. There are doubts about the importance of giving attention to industry involvement in alcohol research, as it is suggested that there are bigger issues to do with research funding and the environment in which alcohol research takes place 128, 130, 143.

**Table 2:** Key discussions on the alcohol industry and science including controversies

Index publication	Nature of the discussion/controversy	Number of other inclusions in this review† and references
Wallack 48	The index publication is a statement of concerns regarding alcohol industry strategies, including some material on science, as well as on policy. Issues discussed in associated records include evidence showing that drinking in moderation may have positive effects on health, and the relationship between the research community and the alcohol industry.	4 134, 138, 140, 150
Edwards *et al*. 28	This is a discussion about ethical issues in science in the addiction field, with some comments about the possible influence exerted by the industry. Associated records are supportive of a policy that would require researchers to disclose their conflicts of interest, and comments were made about the benefits this could have beyond research, including on policy.	4 36, 45, 80, 90
Babor *et al*. 17	This is a discussion and statement of key concerns about the alcohol industry's involvement in alcohol research. Associated records discuss the nature of relationships between researchers and the alcohol industry, and about the links between research and economic and political interests.	9 24, 83, 94, 106, 121, 124, 144, 160, 165
Plant *et al*.^89^	This is a discussion about ethical issues in alcohol research, including the potential influence of funders on researchers, with proposed guidelines and procedures to follow.	2 98, 159
Hannum 131	Dissemination of the Dublin principles of cooperation between the alcohol industry and researchers, developed by a group of experts convened by ICAP and the National College of Industrial Relations of Ireland.	4 132, 152, 153, 154
McCreanor 46	This describes concerns about the strategies used by ICAP, a social aspects organisation funded by the alcohol industry, to influence alcohol policy. Associated records further discuss the involvement of the alcohol industry in science and policy. Perspectives from different countries are presented, and links with other industries are also described.	17 18, 55, 56, 57, 59, 60, 61, 62, 63, 64, 65, 66, 67, 68, 129, 133, 137
Edwards *et al*.^77^	This is a statement of concerns about, the process through which a book was submitted to the journal *Addiction*, where the editor failed to mention that it was funded by the alcohol industry. Two of the related records nuance these concerns. There is also a discussion about the links between ILSI and the tobacco industry.	3 103, 135, 148
James 38	This identifies threats to scientific integrity posed by public private initiatives in which the industry is involved, with a focus on ILSI. Further discussion focuses on the appropriate roles of researchers and the research community, if any, when interacting with the alcohol industry.	2 115, 181
Foxcroft 84	This is an account of the deficiencies in the peer reviewed process for a report produced by ICAP. Associated records discuss the political use of such reports by ICAP, and about its influence on policy more broadly. They also question the nature of relations between researchers and the industry.	4 15, 69, 74, 156
Edwards *et al*. 43	This editorial presents criticism of the appointment of the Chief Executive of the Portman Group to the Board of the Alcohol Education and Research Council by the British government. Associated records further discuss the activities of the Portman Group and the conflicts of interest and influence exerted by the alcohol industry on research and policy.	6 22, 42, 97, 100, 166, 169
Adams 93	This discussion identifies risks taken by public health actors including the research community when receiving money from the dangerous consumption industries, and considers ways to assess these risks. Associated records discuss conflict of interest in science, and the roles that researchers could take in addressing these risks.	4 25, 47, 86, 155
Caetano 51	This presents a critique of a book published by four organisations including ICAP. Associated records explore and debate about the political use of science by industry actors.	2 108, 142
Goozner *et al*.^111^	Offers a common standard for conflict of interest disclosure in addiction journals, with content on the relations between researchers and the alcohol industry. The contents are debated in the associated records. There is also a broader discussion about conflicts of interest in science.	4 30, 35, 127, 179
Stenius and Babor 26	A discussion and statement of concerns about the alcohol industry's involvement in alcohol research, as well as recommendations to minimize risks associated with it. Accompanying discussions of the influence exerted by the alcohol industry on science is provided in the associated records.	6 53, 82, 128, 130, 143, 149
Andreasson and McCambridge 16	A position statement from the International Network on Brief Interventions for Alcohol & Other Drugs, is presented which offers a critical view of research funding by the alcohol industry. Associated records discuss the role that industry could have in research, and its efforts to influence science. Suggestions about alternative sources of funding are also provided.	5 27, 34, 49, 146, 147
Allamani and Beccaria 39	A discussion about conflicts of interest in alcohol research is provided. Associated records discuss ways to prevent influence by alcohol industry actors in science, and provide examples of researchers receiving funds or co‐operating with the industry. There is further discussion about the potential reputational damage for scientists working for or with the industry.	5 29, 40, 41, 54, 70

† Including direct interventions by industry actors. ICAP, International Center for Alcohol Policies; ILSI, International Life Sciences Institute.

Finally, there are articulations of support for the ‘Dublin Principles’ 131, 132, 152, 153, 154, an ICAP initiative that sought to promote partnership working between the research community and industry actors, around the time that the addiction research community was itself developing the Farmington consensus on issues relating to journal publication standards including funding disclosure 123. Some support for such partnerships was found in a survey of the research community 145. Lemmens commented on his participation that; ‘my conclusion at the end of the Dublin meeting was that the industry was not willing to accept any restrictions in their dealing with the research field’ 70. The views investigated here do not analyse industry actor interventions in controversies, which have more specific content and/or offer similar views to those previously described 125, 126, 129, 134, 136, 137, 138, 139, 140, 141, 142, 144.

This review identified no published research studies designed to address issues raised by these opposing views, or in the previous section on transgressions of scientific norms. Please note the following references cited here were not included in the review 72, 80, 112, 113, and also that the following records 51, 155, 156, 157, 158, 159, 160, 161, 162, 163, 164, 165, 166, 167, 168, 169, 170, 171, 172, 173 included in the review did not provide directly cited data.

## Discussion

The major contribution of this study is to clarify the nature of the concerns of the research community and to draw attention to the need for, and inform the detailed content of, further research. We identify three main bodies of concern. Firstly, there clearly is much concern about industry activities at the science/policy interface. Yet, there are few studies of this issue, despite the longevity of the expressions of concern. Secondly, alcohol industry funding as a source of bias in science is remarkably under‐studied. Lastly, there are no dedicated studies of transgressions of scientific norms by industry actors, nor of the controversies that have arisen for more than two decades. The lack of attention to corporate actors within science studies has previously been noted as a major limitation of that literature 174. The purposes and underpinning values of studies of the issues examined here should be explicit to limit scope for accusations of moralism. Methodologically rigorous studies may be capable of contributing to minimising the polarisation that exists in the alcohol research community with high quality empirical data which addresses appropriately the complexity of the issues involved. The need to do so was underscored by the member validation exercise, which suggests also that progress is possible.

It is important to give attention to the limitations of this study. This is a somewhat unusual systematic review, comprising a thematic analysis of documentary data which has been systematically gathered. It is quite possible that we will have missed data that should have been included, partly given the nature of the data being sought. We have also only included material which clearly presents data on the alcohol industry and science, not together with data on other industries or other domains 175. Readers may assess the appropriateness of the analytic procedures and the trustworthiness of the resulting manifest themes directly. We do not elaborate conceptually on the latent thematic material or perform in‐depth discourse analyses of the data. This study is also not designed to give equal coverage of the arguments used, but rather offers a summary of the perspectives of researchers as articulated in the included dataset, and an analysis of these views.

Study findings imply a need to: (i) study the involvement of industry actors in research, and how research is being used in alcohol policy making; (ii) make efforts to protect research integrity from cumulative and multi‐layered forms of bias. ICAP has featured prominently among the concerns reviewed here, and this organisation has now been replaced with the International Alliance for Responsible Drinking (IARD) which makes no claim to be independent of industry interests 176, as did ICAP. IARD claims the Dublin Principles are its own 177, and it might be expected that IARD will similarly seek to influence scientific norms, partly in order to use research as a means of policy influence.

Research community concerns led to the conclusions reached in the CLARION Declaration 71, 178, and also by the international brief interventions network, INEBRIA 16, that alcohol industry funding should be avoided. Industry funding will continue to be made available to scientists, however, and colleagues will continue to accept it. Perhaps more likely so in low and middle income countries where research funding is scarcer, and which are targeted for expansion by the alcohol industry 90, 179.

The challenges identified appear to need to be addressed by agencies including journals, professional societies and universities 18, 179, 180. They also require further study of any beneficial or deleterious consequences of industry involvement in research, including for example divisions within the alcohol research community, and also of measures to address concerns. The need for further research to be complemented by protection of research integrity has been previously identified 181.

Future attention to the alcohol industry and science may benefit from being placed in the context of broader programmes of research on corporations, public health and the science/policy interface, both making contributions to the wider science, and by being informed by it. Similarly, more sociological studies of how competing claims are advanced within the alcohol research community when the actions of corporate actors are implicated and contested may provide a complementary way forward. Multi‐disciplinary collaborations will be needed to achieve these aims 5, 182. Stronger science is the best way forward in response to concerns about corporate subversion of science.

## Supporting information


**Appendix S1.** Search strategy.
