# Supplementary material for: Alcohol industry involvement in science: A systematic review of the perspectives of the alcohol research community
Source: Drug Alcohol Rev. 2018 Jun 13;37(5):565–79. doi: 10.1111/dar.12826 (PMC6055701; doi:10.1111/dar.12826)
Supplement: Supplementary file 1 — Appendix S1. Search strategy. [file DAR-37-565-s001.doc]

# Appendix 1: Search strategy

# Database: Medline (Ovid interface)

1. "Alcohol Education Trust".mp.

2. "Alcohol in Moderation".mp.

3. "Best Bar None".mp.

4. "Community Alcohol Partnerships".mp.

5. Drinkaware.mp.

6. "Gannochy Trust".mp.

7. "Global Alcohol Producers Group".mp.

8. "Licensed Trade charity".mp.

9. ("Mentor UK" or "Mentor International").mp.

10. "National Pub Watch".mp.

11. "portman group".mp.

12. "Robertson Trust".mp.

13. "Vintners Charitable Foundation".mp.

14. "Harm Reduction International".mp.

15. "International Alliance for Responsible Drinking".mp.

16. "sedex global".mp.

17. "Beer and Health Europe".mp.

18. "wine in moderation".mp.

19. "Alcohol Awareness Foundation Ireland".mp.

20. "Amsterdam Group".mp.

21. "Arnoldus Group".mp.

22. "Asociación Gremial Chilena de Empresas de Bebidas Espirituosas Pro Consumo Responsible".mp.

23. "Industry Association for Responsible Alcohol Use".mp.

24. Drinkwise.mp.

25. "Educ’alcool".mp.

26. "Avec moderation!".mp.

27. "Foreningen Gode Alkoholdninger".mp.

28. "Foundation for Advancing Alcohol Responsibility".mp.

29. "Fundacion Alcohol y Sociedad".mp.

30. "Foundacion de Investigaciones Sociales".mp.

31. "Fundación para la investigación del vino y nutrición".mp.

32. "Stichting Verantwoorde Alcoholconsumptie".mp.

33. "God Alkoholkultur".mp.

34. "Hong Kong Forum for Responsible Drinking".mp.

35. "Hungarian Association for Responsible alcohol consumption".mp.

36. "Institut de Recherches Scientifiques sur les Boissons".mp.

37. "Romanian Forum for Responsible Drinking".mp.

38. "Society for Alcohol and Social Policy Initiative ".mp.

39. "Taiwan Beverage Alcohol Forum".mp.

40. "Thai Foundation for Responsible Drinking".mp.

41. "Self-Regulating Alcohol Industry Forum".mp.

42. "Vietnam Association for Responsible Drinking".mp.

43. "Women of Wine charities".mp.

44. "Geary Institute".mp.

45. "Ernest Gallo Clinic and Research Center".mp.

46. "Centro de Informações sobre Saúde e Álcool".mp.

47. "European Foundation for Alcohol Research".mp.

48. "International Life Sciences Institute".mp.

49. "Foundation for Alcohol Related Research".mp.

50. "Korea Alcohol Research Foundation".mp.

51. "Australian Wine Research Institute".mp.

52. "Alcoholic Beverage Medical Research Foundation".mp.

53. "Foundation for Alcohol Research".mp.

54. "Fondation pour la Recherche en Alcoologie".mp.

55. ("Campden Brewing Research International" or "Campden BRI").mp. [mp=title, abstract, original title, name of substance word, subject heading word, keyword heading word, protocol supplementary concept word, rare disease supplementary concept word, unique identifier, synonyms]

56. "The Wine Information Council".mp.

57. "International Scientific Forum on Alcohol Research".mp.

58. "Social Issues Research Centre".mp.

59. "Nuorten Academy".mp.

60. "Aspall Suffolk".mp.

61. "Aston Manor Cider".mp.

62. "Castle Rock Brewery".mp.

63. "Charles Wells".mp.

64. "Edrington Group".mp.

65. "Halewood International".mp.

66. "Hallgarten Druitt".mp.

67. "Ian Macleod Distillers".mp.

68. "JW Lees".mp.

69. "Shepherd Neame".mp.

70. "Nyetimber Product of England".mp.

71. "Tails Cocktails".mp.

72. "Thatchers Cider".mp.

73. "SHS Drinks".mp.

74. "The Somerset Cider Brandy Company".mp.

75. "St Austell Brewery".mp.

76. "William Grant & Sons Limited".mp.

77. "Worshipful company of vintners".mp.

78. "C & C plc".mp.

79. "AB InBev".mp.

80. "Accolade Wines".mp.

81. "Asahi Group Holdings".mp.

82. Bacardi.mp.

83. "Brown-Forman".mp.

84. Calsberg.mp.

85. Diageo.mp.

86. Heineken.mp.

87. "International Beverage Holding Limited".mp.

88. "Kirin Holdings".mp.

89. "Kopparberg".mp.

90. "Molson Coors".mp.

91. "LVMH Moët Hennessy".mp.

92. ("Pernod Ricard" or "Chivas Brothers").mp.

93. "SAB-Miller".mp.

94. Suntory.mp.

95. "Treasury Wine Estates".mp.

96. AMBEV.mp.

97. "Carlton & United Breweries".mp.

98. "Concha y Toro".mp.

99. "Constellation Brands".mp.

100. "Coopers Brewery".mp.

101. "E. & J. Gallo Winery".mp.

102. "Gruppo Campari".mp.

103. "Onebev".mp.

104. "Admiral Taverns".mp.

105. "Amber Taverns".mp.

106. "Drinkwell Bars".mp.

107. "Enterprise Inns".mp.

108. "Greene King".mp.

109. "J D Wetherspoon".mp.

110. "Mittchells and Butlers".mp.

111. "Punch Taverns".mp.

112. "Sankey’s Pub & Seafood Brasserie".mp.

113. "Stonegate Pub Company".mp.

114. "Armit Wines".mp.

115. ASDA.mp.

116. "Cooperative Group".mp.

117. "Conviviality Plc".mp.

118. "Hatch Mansfield".mp.

119. "Wm Morrison".mp.

120. "Negociants UK".mp.

121. "Nisa Retail".mp.

122. Rontect.mp.

123. "Sainsbury’s".mp.

124. "Simply Fresh".mp.

125. Thwaites.mp.

126. Waitrose.mp.

127. Aldi.mp.

128. Lidl.mp.

129. "Marks and Spencer".mp.

130. "Musgave Group".mp.

131. SPAR.mp.

132. Tesco.mp.

133. "Association of Small Direct Wine-Merchants".mp.

134. "British Beer and Pub Association".mp.

135. "British Institute of Inn keeping".mp.

136. "British Sake Association".mp.

137. "Gin and Vodka Association".mp.

138. "National Association of Cider Makers".mp.

139. "Scottish Beer and Pub Association".mp.

140. "Scotch Whisky Association".mp.

141. "Scottish Licensed Trade Association".mp.

142. "Society of Independent Brewers".mp.

143. "Alcohol Beverage Federation of Ireland".mp.

144. "Grocery Manufacturers Association".mp.

145. "International Organisation of Vine and Wine".mp.

146. "International Wine Clubs Association".mp.

147. "International Wine and Spirits Federation".mp.

148. "Wine and Spirit Trade Association".mp.

149. "Worldwide Brewing Alliance".mp.

150. "Wine World Trade group".mp.

151. "Brewers of Europe".mp.

152. "Comité Européen des Entreprises Vins".mp.

153. "Confédération européenne des vignerons indépendants".mp.

154. "European Brands Association".mp.

155. SpiritsEurope.mp.

156. ABRABE.mp.

157. "Alcohol Beverages Australia".mp.

158. "American Beverage Institute".mp.

159. "Associação Nacional de Bebidas Espirituosas".mp.

160. "Beer Canada".mp.

161. "Beer Institute".mp.

162. "Manufacturers Association of Nigeria".mp.

163. "Beer Selling Industry".mp.

164. "Brewers Association of Australia and New Zealand".mp.

165. "Brewers Association of Japan".mp.

166. "Bulgarian Spirits Association".mp.

167. "Cerveceros de México".mp.

168. "Cerveceros Latinoamericanos".mp.

169. "China Alcoholic Drinks Association".mp.

170. "Czech Spirits Association".mp.

171. "Deutsche Weinakademia".mp.

172. "Distilled Spirits Industry Council of Australia".mp.

173. "Distilled Spirits Council of the United States".mp.

174. "French Federation of Spirits Drinks".mp.

175. "International Spirits & Wines Association of India".mp.

176. "Japanese Spirits and Liquor Makers Association".mp.

177. "Korea Alcohol and Liquor Industry Association".mp.

178. "Spanish Federation of Spirits".mp.

179. ("Spanish Wine Federation" or "federacion espanola del vino").mp. [mp=title, abstract, original title, name of substance word, subject heading word, keyword heading word, protocol supplementary concept word, rare disease supplementary concept word, unique identifier, synonyms]

180. "Spirits Canada".mp.

181. "Spirits New Zealand".mp.

182. "Union of Russian Brewers".mp.

183. "Vignerons Independants de France".mp.

184. "Wine Institute".mp.

185. "Association of Convenience Stores".mp.

186. "Association of Licensed Multiple Retailers".mp.

187. "British Hospitality Association".mp.

188. "British Retail Consortium".mp.

189. "Confederation of British Industry".mp.

190. "European Sponsorship Association".mp.

191. "Federation of Licensed Victuallers Associations".mp.

192. "National Federation of Retail Newsagents".mp.

193. "Scottish Grocers Federation".mp.

194. "Scottish Retail Consortium".mp.

195. "UK Travel Retail Forum".mp.

196. "Adam Smith Institute".mp.

197. "Centre for Economics and Business Research".mp.

198. Demos.mp.

199. "Inside media".mp.

200. "Institute of Economic Affairs".mp.

201. "Taxpayers' Alliance".mp.

202. "Institute of Public Affairs Australia".mp.

203. 1 or 2 or 3 or 4 or 5 or 6 or 7 or 8 or 9 or 10 or 11 or 12 or 13 or 14 or 15 or 16 or 17 or 18 or 19 or 20 or 21 or 22 or 23 or 24 or 25 or 26 or 27 or 28 or 29 or 30 or 31 or 32 or 33 or 34 or 35 or 36 or 37 or 38 or 39 or 40 or 41 or 42 or 43 or 44 or 45 or 46 or 47 or 48 or 49 or 50 or 51 or 52 or 53 or 54 or 55 or 56 or 57 or 58 or 59 or 60 or 61 or 62 or 63 or 64 or 65 or 66 or 67 or 68 or 69 or 70 or 71 or 72 or 73 or 74 or 75 or 76 or 77 or 78 or 79 or 80 or 81 or 82 or 83 or 84 or 85 or 86 or 87 or 88 or 89 or 90 or 91 or 92 or 93 or 94 or 95 or 96 or 97 or 98 or 99 or 100 or 101 or 102 or 103 or 104 or 105 or 106 or 107 or 108 or 109 or 110 or 111 or 112 or 113 or 114 or 115 or 116 or 117 or 118 or 119 or 120 or 121 or 122 or 123 or 124 or 125 or 126 or 127 or 128 or 129 or 130 or 131 or 132 or 133 or 134 or 135 or 136 or 137 or 138 or 139 or 140 or 141 or 142 or 143 or 144 or 145 or 146 or 147 or 148 or 149 or 150 or 151 or 152 or 153 or 154 or 155 or 156 or 157 or 158 or 159 or 160 or 161 or 162 or 163 or 164 or 165 or 166 or 167 or 168 or 169 or 170 or 171 or 172 or 173 or 174 or 175 or 176 or 177 or 178 or 179 or 180 or 181 or 182 or 183 or 184 or 185 or 186 or 187 or 188 or 189 or 190 or 191 or 192 or 193 or 194 or 195 or 196 or 197 or 198 or 199 or 200 or 201 or 202

204. corporat*.mp. or exp Industry/ or industr*.mp. or compan*.mp. or business*.mp. or firm*.mp.

205. exp Alcohols/ or alcohol*.mp. or drink*.mp. or exp Alcohol Drinking/

206. 204 and 205

207. 203 or 206

208. doubt.mp.

209. exp Fraud/ or corrupt*.mp.

210. "contract research".mp.

211. non-publication.mp.

212. (ghost-writing or ghostwriting).mp.

213. "junk science".mp.

214. "sound science".mp.

215. ("sponsor* adj2 scien*" or "sponsor* adj2 research*").mp.

216. ("fund* adj2 scien*" or "fund* adj2 research*").mp.

217. ("bend* adj2 science" or "bend* adj2 research" or "bend* adj2 evidence").mp.

218. ("attack* research*" or "attack* scien*").mp.

219. ("manipulat* adj2 science*" or "manipulat* adj2 evidence*" or "manipulat* adj2 data" or "manipulat* adj2 research*").mp.

220. ("undermin* adj2 science" or "undermin* adj2 evidence" or "undermin* adj2 research").mp.

221. "misrepresent* adj2 evidence*".mp.

222. ("distort* adj2 science" or "distort* adj2 evidence" or "distort* adj2 research" or "distort* adj2 data").mp.

223. ("suppress* adj5 research" or "suppress* adj5 evidence" or "suppress* ajd5 science" or "suppress* ajd5 data").mp.

224. cherry#pick*.mp. [mp=title, abstract, original title, name of substance word, subject heading word, keyword heading word, protocol supplementary concept word, rare disease supplementary concept word, unique identifier, synonyms]

225. "non peer#review*".mp. [mp=title, abstract, original title, name of substance word, subject heading word, keyword heading word, protocol supplementary concept word, rare disease supplementary concept word, unique identifier, synonyms]

226. "scientific journal*".mp.

227. ("scientific workshop*" or "scientific seminar*" or "scientific meeting*" or "scientific conference*").mp.

228. exp "Conflict of Interest"/ or "conflict* Adj2 interest*".mp.

229. ("bias* research" or "bias* science" or "bias* evidence").mp.

230. "funding effect*".mp.

231. ("influenc* adj5 science*" or "influenc* adj5 research*").mp. [mp=title, abstract, original title, name of substance word, subject heading word, keyword heading word, protocol supplementary concept word, rare disease supplementary concept word, unique identifier, synonyms]

232. 208 or 209 or 210 or 211 or 212 or 213 or 214 or 215 or 216 or 217 or 218 or 219 or 220 or 221 or 222 or 223 or 224 or 225 or 226 or 227 or 228 or 229 or 230 or 231

233. 207 and 232

234. limit 233 to yr="1980 - 2016"

Results= 135
